# The relationship between serum fatty-acid binding protein 4 level and lung function in Korean subjects with normal ventilatory function

**DOI:** 10.1186/s12890-016-0190-8

**Published:** 2016-02-18

**Authors:** Hye-Jeong Park, Se Eun Park, Cheol-Young Park, Seong Yong Lim, Won-Young Lee, Ki-Won Oh, Sung-Woo Park, Eun-Jung Rhee

**Affiliations:** Department of Endocrinology and Metabolism, Kangbuk Samsung Hospital, Sungkyunkwan University School of Medicine, Seoul, Korea; Division of Pulmonary and Critical Care Medicine, Department of Internal Medicine, Kangbuk Samsung Hospital, Sungkyunkwan University School of Medicine, Seoul, Korea

**Keywords:** Fatty-acid binding protein-4, Ventilatory function, Adipokine

## Abstract

**Background:**

The aim of this study was to assess the association of lung function with serum fatty-acid binding protein 4 (FABP4) in apparently healthy Korean adults.

**Methods:**

In 495 participants in a health screening program, Force Exploratory Volume (FEV) 1 and Forced Vital Capacity (FVC) were assessed with standard spirometry. Subjects with obstructive (*n* = 19) and restrictive (*n* = 45) lung function were excluded from the analysis. Serum FABP4 level was measured by enzyme-linked immunosorbent assay and transformed into Ln(FABP4). 431 subjects with normal ventilator function (72.4 % men, mean age 41 years) were included in the final analysis.

**Results:**

Mean Ln(FABP4) significantly decreased in subjects from 1^st^ quartile to 4^th^ quartile of FVC (*p* = 0.008). Ln(FABP4) did not show significant differences across the quartile groups of FEV1. The odds ratio (OR) of being in the lowest quartile of FVC was 2.704 in subject with 3^rd^ tertile of Ln(FABP4) after full adjustment for confounding variables {95 % confidence interval (CI) 1.397 ~ 5.357}. OR of being in the lowest quartile of FEV1 was 1.822 (95 % CI 1.021 ~ 3.298) in subjects with 3^rd^ tertile of Ln(FABP4) after adjustment of age and sex, which was attenuated after full adjustment for confounding variables.

**Conclusion:**

Increased FABP4 level showed increased risk for reduced lung function in subjects with normal ventilatory function.

**Electronic supplementary material:**

The online version of this article (doi:10.1186/s12890-016-0190-8) contains supplementary material, which is available to authorized users.

## Background

Adipokines, such as leptin, adiponectin, ghrelin, resistin, and visfatin, are peptides that are secreted from visceral adipose tissues [1]. Adipocytokines secreted from adipose tissue are the results of close interactions between adipocytes and immune cells that are infiltrated in adipose tissue. Adipocytokines mediate the crosstalk between adipose tissues and other metabolic organs in our body, especially the liver, muscle, pancreas, and the organs of the central nervous system. They maintain metabolic homeostasis and their dysfunctions have been causally linked to a wide range of metabolic diseases [2–4].

Adipocytokines are known to play a role in the process of development of lung diseases from previous studies [5]. Ghrelin level in circulation is decreased in patients with chronic obstructive pulmonary disease [6]. Visfatin—initially known as pre-B cell colony-enhancing factor—also plays a critical role in some inflammatory processes, the apoptosis of neutrophils, and the secretion of interleukin-8 from the endothelial cells of the pulmonary artery in humans [7]. Furthermore, decreased forced vital capacity (FVC) or forced expiratory volume in one second (FEV1), indicating impaired lung function, is associated with persistent low grade systemic inflammation assessed by serum inflammatory markers, such as C-reactive protein (CRP) [8, 9].

Although emerging evidence has shown that pulmonary dysfunction has a connection with not only cigarette smoking, but also with obesity, type 2 diabetes, and insulin resistance [9–12], only few studies have investigated the relationship between reduced lung function and adipocytokine levels. Fatty acid–binding protein 4 (FABP4) is an adipocytokine that is expressed in both adipocytes and macrophages [13]. It is known that the FABP4 level is associated with obesity, insulin resistance, and atherosclerosis. It has been reported that FABP4 acts as a positive regulator of cellular proliferation; therefore its role in lung function may be important [14]. However, little is known about the relationship between reduced lung function and FABP4 levels.

We therefore assessed the association between increased serum FABP4 levels with reduced lung function in apparently healthy Korean adults with normal ventilatory function.

## Methods

### Study subjects

This study was performed as a sub-study in the Kangbuk Samsung Medical Center (KBSMC)-adipokine study, which is a longitudinal study performed in subjects who received annual health check-ups at the Health Promotion Center at Kangbuk Samsung Hospital in Seoul, Korea, between 2003 and 2007. In 1635 serum samples of the subjects who participated in annual health check-up in 2003, 499 subjects in whom serum adipocytokine levels were available, were selected to participate in this study. Of them, 4 individuals were excluded because they had no available FEV1 or FVC level measurements. In total of 495 subjects, 45 subjects (9.1 %) had restrictive ventilatory pattern and 19 subjects (3.8 %) had obstructive pattern. The final analyses were performed in 431 subjects with normal ventilatory function after exclusion of these 64 subjects.

The study protocol conformed to ethical guidelines of the 2000 Declaration of Helsinki, and accordingly the Kangbuk Samsung Hospital Human Research Committee approved it. The Kangbuk Samsung Hospital Institutional Review Board also approved this study. The informed consent requirement for this study was exempted by the Institutional Review Board at the time the study was in the planning phase because researchers only accessed the database for analysis purposes, which was free of identifying personal information.

### Measurement of lung function

Spirometry was performed as recommended by the American Thoracic Society, using Vmax22 (SensorMedics, Yorba Linda, CA, USA). Absolute Values of FVC and FEV1 were obtained and the percentage of predicted values (% pred) for FEV1 and FVC were calculated from the following equations obtained in a representative Korean Population [15].

$$ \mathrm{Predicted}\ \mathrm{F}\mathrm{V}\mathrm{C} = 4.8434\ \hbox{--} \kern0.5em \left(0.00008633 \times \mathrm{ag}{\mathrm{e}}^2\left[\mathrm{years}\right]\right) + \left(0.05292 \times \mathrm{height}\ \left[\mathrm{cm}\right]\right) + \left(0.01095 \times \mathrm{weight}\ \left[\mathrm{kg}\right]\right) $$

$$ \mathrm{Predicted}\ \mathrm{F}\mathrm{E}\mathrm{V}1 = 3.4132\ \hbox{--}\ \left(0.0002484 \times \mathrm{ag}{\mathrm{e}}^2\left[\mathrm{years}\right]\right) + \left(0.04578 \times \mathrm{height}\ \left[\mathrm{cm}\right]\right) $$

The highest FVC and FEV1 values of the tests with acceptable curves were used. Ventilatory patterns were defined as normal (FVC ≥80 % and FEV1/FVC ≥0.7), restrictive (FVC <80 % and FEV1/FVC ≥0.7; *n* = 45; 9.1 %), or obstructive (FEV1/FVC <0.7; *n* = 19; 3.8 %).

Subjects with normal patterns were subdivided into quartiles according to the baseline percentage of predicted values (% predicted) for FVC or FEV1. Based on the FVC, the resulting four categories were as follows: ≤99.3 % in quartile 1, 99.3–104.4 % in quartile 2, 104.4–112.7 % in quartile 3, and >112.7 % in quartile 4. Similarly, the subjects were also divided into quartiles based on the FEV1 values (% predicted): ≤97.9 % in quartile 1, 97.9–106.9 % in quartile 2, 106.9–118.2 % in quartile 3, and >118.2 % in quartile 4.

### Anthropometric measurements and laboratory tests

Trained physicians carried out the anthropometric measurements including height, weight, and body fat percentage. Systolic (SBP) and diastolic blood pressures (DBP) were measured according to the Hypertension Detection and Follow-up Program protocol by using a mercury blood pressure device after the subjects had rested longer than 5 min [16]. For the cases with a SBP higher than 140 mmHg and a DBP higher than 90 mmHg, the BP was measured two more times after resting and the average value was used. Body mass index (BMI) (kg/m^2^) was calculated by dividing the weight (kg) by the squared height (m).

After 12 h of fasting, blood glucose (FBG), total cholesterol (TC), triglyceride (TG), high-density lipoprotein cholesterol (HDL-C), and low-density lipoprotein-cholesterol (LDL-C) levels were obtained. The hexokinase method (Advia1650 Autoanalyzer; Bayer Diagnostics, Leverkusen, Germany) was used to measure blood glucose levels and an enzymatic colorimetric test was used to measure TC and TG levels. The selective inhibition method was used to measure HDL-C and a homogeneous enzymatic colorimetric test was used to measure LDL-C. The fasting serum insulin level was measured by immunoradiometric assay (RIABEAD II, Abbott, Tokyo, Japan), with an intra-assay coefficient of variance of 1.2 to 1.9 % and an inter-assay coefficient of variance of 1.4 to 3.3 %. The results were presented as milligrams per liter and the limit of measurement was 0.175 mg/L, with a sample dilution of 1:20. Insulin resistance status was calculated by using the homeostatic model assessment-insulin resistance (HOMA-IR) [17]. The formula used for calculation was as follows:

$$ \mathrm{HOMA}\hbox{-} \mathrm{I}\mathrm{R} = \left(\mathrm{fasting}\ \mathrm{insulin}\ \left[\upmu \mathrm{I}\mathrm{U}/\mathrm{mL}\left] \times \mathrm{fasting}\ \mathrm{blood}\ \mathrm{glucose}\ \right[\mathrm{m}\mathrm{M}/\mathrm{L}\right]\right)/22.5. $$

### Measurement of serum FABP4 levels

Serum FABP4 levels were measured using an enzyme-linked immunosorbent assay (ELISA; BioVendor Laboratory Medicine, Modrice, The Czech Republic). The intra- and inter-assay coefficients of variation (CVs) were 5.0 and 9.8 %, respectively.

Subjects were divided into three groups according to the Ln(FABP4); 1^st^ tertile: Ln(FABP4) < 2.04, 2^nd^ tertile: 2.04 ≤ Ln(FABP4) < 2.41, 3^rd^ tertile: Ln(FABP4) ≥ 2.41.

### Evaluation of body composition

Body composition measurements of the subjects were carried out by segmental bioelectric impedance, using eight tractile electrodes according to the manufacturer’s instructions (InBody 3•0, Biospace, Korea). Lean mass (kg), fat mass (kg), percent fat mass (%), and waist-hip ratio (WHR) as a marker of abdominal obesity, were measured.

### Statistical analysis

Statistical analysis was performed with SPSS version 18.0 (PASW Statistics version 18.0 (SPSS Inc., Chicago, IL, USA). Data are represented as mean descriptive statistics and were used to describe the mean ± SD. Descriptive statistics were used to describe the study population at the baseline. The assessment for the normality of the variables was done with Kormogorov–Smirnov test and the FABP4 values were log-transformed as they did not follow a normal distribution. The comparisons of mean values of the variables among the subdivided groups were performed by Kruskal-Wallis test or one-way ANOVA and multiple comparison tests were performed with post hoc analysis. The chi-square test was used for cross-tabulation analysis. Stepwise multiple logistic regression analyses were performed to evaluate the associations between FABP4 and being in the lowest FVC and FEV1 (% pred) quartiles after adjustment for confounding factors. A 95 % confidence interval (CI) was determined for each risk. For all statistical tests used, a *p* value <0.05 was considered significant.

## Results

### Clinical characteristics of total study participants according to the ventilatory pattern

Clinical characteristics of the total study population based on the ventilatory patterns are presented in Table 1. 431 (87.1 %) subjects had normal ventilator pattern, 45 (9.1 %) had restrictive ventilatory pattern and 19 (3.8 %) subjects had obstructive ventilatory pattern. Mean age of the participants was 41 years. Subjects with restrictive ventilator pattern were younger and leaner than subjects with normal ventilatory pattern. The subjects with obstructive ventilatory pattern showed similar metabolic profiles to subjects with normal ventilatory pattern. There were no significant differences in FABP4 levels among the three groups.

**Table 1:** Baseline characteristics of total population

	All (*N* = 495)	Normal (*N* = 431)	Restrictive (*N* = 45)	Obstructive (*N* = 19)	*P value*
Age (yrs)	40.83 ± 6.27	41.10 ± 6.30^a^	38.96 ± 5.95^b^	39.05 ± 5.46^a,b^	0.041
Sex: male (%)	327 (66.1)	312 (72.4)	7 (15.6)	8 (42.1)	<0.001
BMI (kg/m^2^)	23.63 ± 2.96	23.90 ± 2.92^a^	21.50 ± 2.63^b^	22.59 ± 2.42^a,b^	<0.001
SBP (mmHg)	112.91 ± 12.21	113.48 ± 12.03^a^	106.67 ± 11.68^b^	114.74 ± 13.89^a,c^	0.001
DBP (mmHg)	72.67 ± 9.51	73.11 ± 9.40^a^	68.00 ± 9.19^b^	73.68 ± 10.12^a,c^	0.002
Fasting glucose (mg/dL)	94.40 ± 12.08	94.88 ± 12.40	90.84 ± 10.34	92.05 ± 5.50	0.071
Total cholesterol (mg/dL)	200.89 ± 35.72	203.34 ± 36.05^a^	182.53 ± 27.36^b^	188.74 ± 31.66^a,b^	<0.001
Triglyceride (mg/dL)	132.75 ± 111.83	139.19 ± 117.52^a^	87.13 ± 40.43^b^	94.74 ± 40.30^a,b^	0.004
HDL-C (mg/dL)	54.21 ± 11.23	53.88 ± 11.31	57.07 ± 10.18	54.95 ± 11.27	0.185
LDL-C (mg/dL)	114.44 ± 28.11	115.91 ± 28.08^a^	102.54 ± 26.34^b^	105.84 ± 26.25^a,b^	0.008
Fasting insulin (uIU/mL)	6.72 ± 3.45	6.80 ± 3.56^a^	6.74 ± 2.64^a,b^	4.83 ± 1.89^c^	0.051
Percent body fat (%)	23.55 ± 5.47	23.26 ± 5.46^a^	26.04 ± 4.80^b^	24.27 ± 5.94^a,b^	0.004
WHR	0.86 ± 0.05	0.86 ± 0.05^a^	0.83 ± 0.04^b^	0.85 ± 0.04^a,b^	<0.001
HOMA-IR	1.58 ± 0.94	1.60 ± 0.96	1.55 ± 0.81	1.11 ± 0.46	0.076
FVC (% pred)	1.01 ± 0.16	1.04 ± 0.13^a^	0.71 ± 0.08^b^	1.02 ± 0.16^b,c^	<0.001
FEV1 (% pred)	1.05 ± 0.19	1.09 ± 0.16^a^	0.75 ± 0.10^b^	0.76 ± 0.12^b^	<0.001
Ln(FABP4)	2.21 ± 0.45	2.22 ± 0.45	2.18 ± 0.38	2.10 ± 0.52	0.490
Smoking (%)	80 (16.2)	78 (18.1)	1 (2.2)	1 (5.3)	0.009

Different superscripts denote a significant difference between groups

Values were expressed as Mean ± SD or N (%)

*BMI* body mass index, *SBP* systolic blood pressure, *DBP* diastolic blood pressure, *HDL-C* high-density lipoprotein cholesterol, *LDL-C* low-density lipoprotein cholesterol, *WHR* waist-hip ratio, *HOMA-IR* homeostasis model assessment-insulin resistance, *FVC* force vital capacity, *FEV1* forced expiratory volume in 1 s, *Ln(FABP4)* logarithmized form of fatty-acid binding protein 4

Further analyses were performed only in subjects with normal ventilatory function (*N* = 431).

### Comparisons of metabolic components by quartiles of FVC and FEV1 (% pred) in subjects with normal ventilatory function

**Fig. 1:**
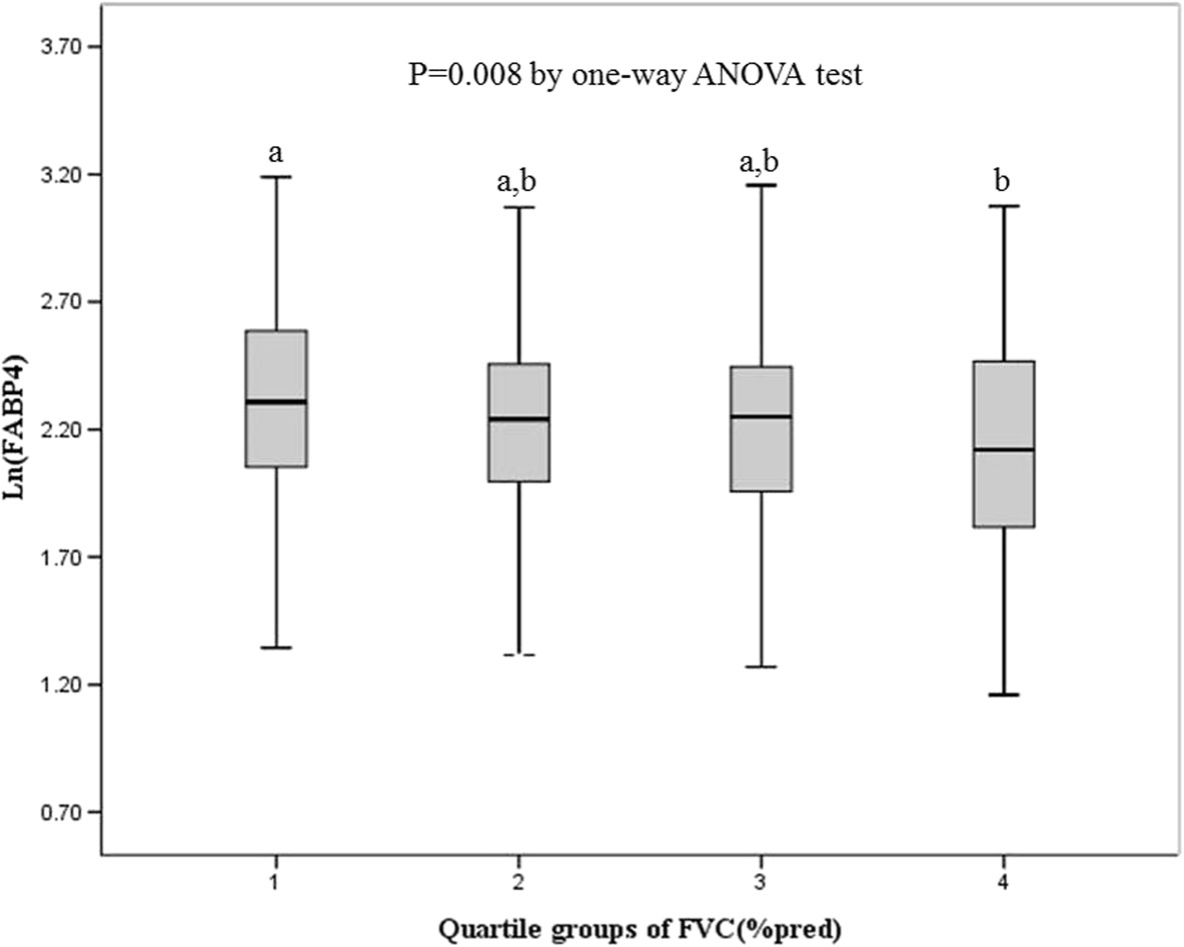
Comparisons of mean Ln(FABP4) among the quartile groups of FVC (% pred) in subjects with normal lung function

Mean age of the subjects with normal ventilatory function was 41 years and 312 subjects (72.4 %) were men. When the mean values of metabolic parameters were compared according to the quartiles of FVC (% pred), age, HDL-C and percent body fat showed significant differences among the groups (Table 2). The mean values of HDL-C and percent body fat decreased from quartile 1 to quartile 4. Mean Ln(FABP4) significantly decreased from quartile 1 to quartile 4 of FVC (% pred) (Table 2, Fig. 1).

**Table 2:** Comparisons of metabolic components by quartile of FVC (% pred) among subjects with normal ventilator function

*N* = 431	1^st^ Quartile	2^nd^ Quartile	3^rd^ Quartile	4^th^ Quartile	*P value*
	(≤0.993)	(>0.993, ≤ 1.044)	(>1.044, ≤ 1.127)	(>1.127)	
Age (yrs)	42.06 ± 6.4^a^	42.42 ± 7.2^a^	39.32 ± 5.0^b^	40.61 ± 6.0^a,b^	0.001
Sex: male (%)	58 (53.7)^a^	84 (77.8)^b^	79 (73.2)^b^	91 (84.3)^b^	<0.001
BMI (kg/m^2^)	23.62 ± 3.3	24.15 ± 3.1	23.84 ± 2.7	23.98 ± 2.5	0.595
SBP (mmHg)	110.83 ± 13.3	113.7 ± 12.0	114.81 ± 11.3	114.58 ± 11.0	0.057
DBP (mmHg)	71.67 ± 10.3	73.43 ± 9.1	73.8 ± 9.7	73.55 ± 8.4	0.325
Fasting glucose (mg/dL)	94.06 ± 11.9	95.84 ± 15.0	94.46 ± 8.8	95.15 ± 13.2	0.733
Total cholesterol (mg/dL)	206.54 ± 40.6	204.56 ± 31.2	202.79 ± 36.2	199.43 ± 35.7	0.522
TG (mg/dL)	140.83 ± 166.7	145.21 ± 121.4	140.4 ± 94.6	130.21 ± 62.7	0.817
HDL-C (mg/dL)	56.41 ± 13.4^a^	53.48 ± 9.7^a,b^	52.35 ± 11.5^b^	53.27 ± 10.0^a,b^	0.049
LDL-C (mg/dL)	117.16 ± 30.8	116.2 ± 25.1	117.33 ± 27.6	112.86 ± 29.0	0.650
Fasting insulin (uIU/mL)	7.21 ± 4.3	6.61 ± 3.1	6.79 ± 3.6	6.61 ± 3.1	0.571
Percent body fat (%)	24.90 ± 5.8^a^	23.16 ± 5.8^a,b^	22.93 ± 4.7^b^	22.02 ± 5.2^b^	0.001
WHR	0.86 ± 0.1	0.87 ± 0.1	0.86 ± 0.04	0.87 ± 0.04	0.448
HOMA-IR	1.72 ± 1.3	1.55 ± 0.8	1.6 ± 0.9	1.55 ± 0.8	0.537
Ln(FABP4)	2.34 ± 0.5^a^	2.21 ± 0.5^a,b^	2.19 ± 0.4^a,b^	2.13 ± 0.5^b^	0.008
Smoking (%)	12 (11.2)	20 (18.7)	20 (18.7)	26 (24.3)	0.095

Different superscripts denote a significant difference between groups

Values were expressed as Mean ± SD or N (%)

*FVC* forced vital capacity, *BMI* body mass index, *SBP* systolic blood pressure, *DBP* diastolic blood pressure, *TG* triglyceride, *HDL-C* high-density lipoprotein cholesterol, *LDL-C* low-density lipoprotein cholesterol, *WHR* waist-hip ratio, *HOMA-IR* homeostasis model assessment-insulin resistance, *Ln(FABP4)* logarithmized form of fatty-acid binding protein 4

When the mean values of metabolic parameters were compared according to the quartiles of FEV1 (% pred), mean values of BMI, BP, HDL-C, percent body fat and WHR showed significant differences among the groups (Table 3). The mean values of BMI and BP increased as the quartiles of FEV1 (% pred) increased from quartile 1 to quartile 4. Mean HDL-C values decreased from quartile 1 to quartile 4. Other components of metabolic syndrome showed inconsistent results. The mean percent body fat of 1^st^ quartile was significantly lower than other quartile groups (*p* < 0.001). The mean Ln(FABP4) values did not show significant differences across the quartile groups.

**Table 3:** Comparisons of metabolic components by quartile of FEV1 (% pred) among subjects with normal ventilator function

*N* = 431	1^st^ Quartile (≤0.979)	2^nd^ Quartile (>0.979, ≤ 1.069)	3^rd^ Quartile (>1.069, ≤ 1.182)	4^th^ Quartile (>1.182)	*P value*
Age (yrs)	41.44 ± 6.2	40.02 ± 5.5	41.09 ± 6.6	41.88 ± 6.9	0.163
Sex: male (%)	50 (46.3)^a^	77 (71.3)^b^	86 (79.6)^b,c^	99 (91.7)^c^	<0.001
BMI (kg/m^2^)	23.66 ± 3.4^a,b^	23.51 ± 2.7^a^	23.81 ± 2.8^a,b^	24.64 ± 2.6^b^	0.021
SBP (mmHg)	109.54 ± 12.3^a^	113.52 ± 12.9^a,b^	114.63 ± 11.4^b^	116.26^b^ ± 10.5^b^	<0.001
DBP (mmHg)	70.83 ± 10.1^a^	72.59 ± 9.5^a,b^	73.98 ± 9.2^a,b^	75.05 ± 8.4^b^	0.007
Fasting glucose (mg/dL)	93.69 ± 10.6	93.74 ± 8.9	97.05 ± 15.0	95.05 ± 13.9	0.156
Total cholesterol (mg/dL)	207.75 ± 37.0	198.38 ± 35.0	203.19 ± 35.1	204.04 ± 36.9	0.296
TG (mg/dL)	123.58 ± 159.9	135.86 ± 92.8	142.41 ± 91.9	155.04 ± 111.8	0.260
HDL-C (mg/dL)	57.07^a^ ± 11.6	53.03^b^ ± 12.5	53.29^ab^ ± 11.1	52.11^b^ ± 9.2	0.007
LDL-C (mg/dL)	119 ± 27.5	112.38 ± 27.9	116.16 ± 27.5	116.26 ± 29.5	0.411
Fasting insulin (uIU/mL)	6.87 ± 4.1	6.2 ± 2.8	6.88 ± 3.5	7.28 ± 3.7	0.169
Percent body fat (%)	25.73 ± 5.4^a^	22.90 ± 5.4^b^	22.55 ± 5.6^b^	21.84 ± 4.5^b^	<0.001
WHR	0.86 ± 0.1^a,b^	0.86 ± 0.04^a^	0.86 ± 0.04^a,b^	0.88 ± 0.04^b^	0.020
HOMA-IR	1.62 ± 1.2	1.45 ± 0.7	1.65 ± 1.0	1.7 ± 0.9	0.248
Ln(FABP4)	2.29 ± 0.5	2.2 ± 0.4	2.19 ± 0.5	2.19 ± 0.4	0.351
Smoking (%)	13 (12.1)	21 (19.6)	20 (18.7)	24 (22.4)	0.254

Different superscripts denote a significant difference between groups

Values were expressed as Mean ± SD or N (%)

*FEV1* forced expiratory volume in 1 s, *BMI* body mass index, *SBP* systolic blood pressure, *DBP* diastolic blood pressure, *HDL-C* high-density lipoprotein cholesterol, *LDL-C* low-density lipoprotein cholesterol, *WHR* waist-hip ratio, *HOMA-IR* homeostasis model assessment-insulin resistance, *Ln(FABP4)* logarithmized form of fatty-acid binding protein 4

When the mean values of the metabolic parameters were compared between the lowest quartile and other quartile groups divided by FVC (% pred), mean value of SBP were significantly lower, and mean values of HDL-C and percent body fat were significantly higher in the lowest quartile compared to other quartile groups (Additional file 1: Table S1). Mean Ln(FABP4) was significantly higher in the lowest quartile group compared with other quartile groups. Similar results were observed when the comparisons were performed in groups divided by FEV1 (% pred), except that mean Ln(FABP4) showed only a higher tendency in the lowest quartile compared with other quartile groups (*p* = 0.0710) (Additional file 1: Table S2).

### Multiple logistic regression analyses for Ln(FABP4) with lowest quartiles of FVC and FEV1 (% pred) as the dependent variable in subjects with normal ventilatory function

Stepwise multiple logistic regression analyses were performed with being in the lowest quartile of FVC (% pred) as the dependent variable (Table 4). The odds ratio (OR) of being in the lowest quartile of FVC (% pred) was 2.193 in subjects with 3^rd^ tertile of Ln(FABP4) after adjustment for age and sex with the 1^st^ tertile of Ln(FABP4) as the reference {95 % CI 1.239 ~ 3.949}. When the analysis was performed with full adjustment for confounding variables, OR for being in the lowest quartile of FVC (% pred) was 2.704 in subjects with 3^rd^ tertile of Ln(FABP4) (95 % CI 1.397 ~ 5.357).

**Table 4:** Multiple logistic regression analyses with lowest quartile of FVC(% pred) as dependent variable among subjects with normal ventilator function

	OR	95 % CI
Model 1		
Ln(FABP4), 1^st^ tertile	1	–
Ln(FABP4), 2^nd^ tertile	1.736	0.969 ~ 3.154
Ln(FABP4), 3^rd^ tertile	2.193	1.239 ~ 3.949
Model 2		
Ln(FABP4), 1^st^ tertile	1	–
Ln(FABP4), 2^nd^ tertile	1.817	1.002 ~ 3.340
Ln(FABP4), 3^rd^ tertile	2.337	1.251 ~ 4.451
Model 3		
Ln(FABP4), 1^st^ tertile	1	–
Ln(FABP4), 2^nd^ tertile	2.034	1.093 ~ 3.845
Ln(FABP4), 3^rd^ tertile	2.704	1.397 ~ 5.357

Model 1, adjusted for covariates with age, sex; Model 2, adjusted as Model 1 plus significant covariables (smoking, SBP, HDL-C, percent body fat); Model 3, fully adjusted as Model 2 plus insignificant covariables (fasting glucose, DBP, TG, LDL-C, fasting insulin, WHR, HOMA-IR, BMI)

*FVC* forced vital capacity, *OR* odds ratio, *CI* confidence interval, *Ln(FABP4)* logarithmized form of fatty-acid binding protein 4, *SBP* systolic blood pressure, *HDL-C* high-density lipoprotein cholesterol, *DBP* diastolic blood pressure, *TG* triglyceride, *LDL-C* low-density lipoprotein cholesterol, *WHR* waist-hip ratio, *HOMA-IR* homeostasis model assessment-insulin resistance; BMI, body mass index

Similar analyses were performed with being in the lowest quartile of FEV1(% pred) as the dependent variable (Table 5). OR of being in the lowest quartile of FEV1 (% pred) was 1.822 in subjects with 3^rd^ tertile of Ln(FABP4) after adjustment for age and sex. However, further adjustment for confounding variables attenuated the significance (Table 5).

**Table 5:** Multiple logistic regression analyses with lowest quartile of FEV1(% pred) as dependent variable among subjects with normal ventilatory function

	OR	95 % CI
Model 1		
Ln(FABP4), 1^st^ tertile	1	–
Ln(FABP4), 2^nd^ tertile	1.547	0.858 ~ 2.819
Ln(FABP4), 3^rd^ tertile	1.822	1.021 ~ 3.298
Model 2		
Ln(FABP4), 1^st^ tertile	1	–
Ln(FABP4), 2^nd^ tertile	1.406	0.767 ~ 2.597
Ln(FABP4), 3^rd^ tertile	1.579	0.840 ~ 3.002
Model 3		
Ln(FABP4), 1^st^ tertile	1	–
Ln(FABP4), 2^nd^ tertile	1.512	0.816 ~ 2.827
Ln(FABP4), 3^rd^ tertile	1.574	0.819 ~ 3.056

Model 1, adjusted for covariates with age, sex; Model 2, adjusted as Model 1 plus significant covariables (SBP, DBP, HDL-C, body fat percentage); Model 3, fully adjusted as Model 2 plus insignificant covariables (smoking, glucose, TG, fasting insulin, percentage body fat, WHR, HOMA-IR, BMI)

*FEV1* forced expiratory volume in 1 s, *OR* odds ratio, *CI* confidence interval, *Ln(FABP4)* logarithmized form of fatty-acid binding protein 4, *SBP* systolic blood pressure, *DBP* diastolic blood pressure, *HDL-C* high-density lipoprotein cholesterol, *TG* triglyceride, *WHR* waist-hip ratio, *HOMA-IR* homeostasis model assessment–insulin resistance, *BMI* body mass index

## Discussion

Our study is the first study that revealed the association between FABP4 and lung function. The major finding of this study was that the concentration of serum FAPB4 increased as the lung function decreased within the normal range of lung function, after adjustment for a wide range of variables, including age, gender, current smoking, BP, FBG, BMI, HOMA-IR, lipid profile, and percent body fat, when the study population was divided into quartiles based on FVC or FEV1 (% pred). Multiple logistic regression and stepwise logistic regression revealed that increased serum FABP4 correlated with an increased risk for reduced lung function, especially showing significant correlation with FVC.

Impaired lung function is known to be associated with an increased prevalence and mortality of cardiovascular disease. It is well recognized that severe obesity is associated with impaired lung function [18, 19]. Most population studies that examined the relationship between obesity and lung function used BMI as a measurement of overall adiposity and insignificant or weak associations have been reported, with diminished lung function at both extremes of the BMI distribution [20]. Recently, it was suggested that fat mass, and fat-free mass might have distinct effects on pulmonary function. Several studies reported inverse associations between lung function and measures of central adiposity, such as the waist circumference and the WHR [21–23]. Although recent studies suggest the association of impaired lung function and metabolic syndrome with total body fat [24], there have been few studies investigating the relationship between lung function and the related markers.

Adipocytokines are a group of hormone-like mediators secreted by adipose tissues. They were first described as regulators of energy metabolism, but were later also recognized as being produced by inflammatory cells and being involved in many immune and inflammatory processes in the human body [1]. Recently, adipocytokines have also been found to mediate inflammatory responses in the human lung, and associations between the levels of some adipocytokines and obstructive airway diseases have been described [4–6]. It is not well-known that obesity may play a significant role in the pathogenesis of pulmonary diseases through mechanisms in association with pro-inflammatory mediators produced from adipose tissues, and they contribute to a low-grade systemic inflammation. In animal models, inflammatory responses in the lung have been shown to influence the production of adipocytokines, leptin and adiponectin, cytokines, acute phase proteins, and other mediators produced by adipose tissues, which may participate in immune responses in lungs [5, 25, 26]. An increased adipose tissue mass may also influence susceptibility to pulmonary infections, enhance pulmonary inflammation associated with environmental exposures, and exacerbate airway obstruction in preexisting lung diseases [27].

In our study, the FABP level increased as the FVC (% pred) decreased within the normal range of lung function after adjustment for other variables. FABP4 belongs to a family of cytosolic chaperones that are involved in systemic regulation of lipid and glucose metabolism [13]. FABP4 has been implicated in several aspects of the metabolic syndrome in mice, including insulin resistance and atherosclerosis [28–32]. The level of circulating human FABP4 was proposed as an independent prognostic marker for the development of metabolic syndrome, non-alcoholic fatty liver and diabetes [33–36]. To date, the mechanism by which FABP4 promotes insulin resistance, inflammation or pulmonary dysfunction are not fully understood. Decreased FVC or FEV1 has been shown to be persistent in low-grade systemic inflammatory status assessed by increased serum levels of inflammatory markers, such as CRP [8, 9, 37, 38]. Our results imply that FABP4 might also act as a pro-inflammatory adipocytokine and affect pulmonary function. Further research is needed to clarify the role of FABP4 on pulmonary function or inflammation.

Our study has a few limitations. First, our study is cross-sectional study; therefore, we cannot know the cause-effect relationship between FABP4 levels and lung function. Second, the correlation of serum FABP4 and lung function is limited to the FVC (% pred). The reason why FABP4 level did not show significant correlation with FEV1 could not be clarified. Third, as the analyses were performed regarding the relationship only between the FABP4 levels and lung function, the possible effects of other adipocytokines on FABP4 and lung function could not be separately analyzed. In addition, it could be interesting to see other FABPs such as FABP5 and FABP1 in relation to lung function in future studies. Despite these limitations, our study has strength in that this is the first study that analyzed the relationship between FABP4 and lung function in apparently healthy adults.

## Conclusions

In summary, the results of this study indicate that increased FABP4 is related with reduced lung function, especially decreased FVC (% pred) even after adjustment for confounding factors in subjects with normal lung function. Further prospective studies are needed to clarify the effect of FABP4 on lung function.

## Additional file

Additional file 1: Table S1. Comparisons of the metabolic components in the lowest quartile and other quartiles of FVC (% pred) among subjects with normal ventilatory function. **Table S2.** Comparisons of the metabolic components in the lowest quartile and other quartiles of FEV1 (% pred) among subjects with normal ventilatory function. (DOCX 18 kb)

## Abbreviations

BMI: Body mass index
CI: Confidence interval
CRP: C-reactive protein
DBP: Diastolic blood pressure
FABP4: Fatty acid–binding protein 4
FBG: Fasting blood glucose
FEV1: Forced expiratory volume in one second
FVC: Forced vital capacity
HDL-C: High-density lipoprotein cholesterol
HOMA-IR: Homeostasis model assessment-insulin resistance
LDL-C: Low-density lipoprotein cholesterol
SBP: Systolic blood pressure
TC: Total cholesterol
TG: Triglyceride
WHR: Waist-hip ratio

## References

[1] Cao H (2014). Adipocytokines in obesity and metabolic disease. J Endocrinol.

[2] Ouchi N, Kihara S, Funahashi T, Matsuzawa Y, Walsh K (2003). Obesity, adiponectin and vascular inflammatory disease. Curr Opin Lipidol.

[3] Ouchi N, Ohashi K, Shibata R, Murohara T (2012). Adipocytokines and obesity-linked disorders. Nagoya J Med Sci.

[4] Ouchi N, Parker JL, Lugus JJ, Walsh K (2001). Adipokines in inflammation and metabolic disease. Nat Rev Immunol.

[5] Shore SA (2006). Obesity and asthma: cause for concern. Curr Opin Pharmacol.

[6] Luo FM, Liu XJ, Li SQ, Wang ZL, Liu CT, Yuan YM (2005). Circulating ghrelin in patients with chronic obstructive pulmonary disease. Nutrition.

[7] Jia SH, Li Y, Parodo J, Kapus A, Fan L, Rotstein OD (2004). Pre-B cell colony-enhancing factor inhibits neutrophil apoptosis in experimental inflammation and clinical sepsis. J Clin Invest.

[8] Lawlor DA, Ebrahim S, Smith GD (2004). Associations of measures of lung function with insulin resistance and Type 2 diabetes: findings from the British Women’s Heart and Health Study. Diabetologia.

[9] Lim SY, Rhee EJ, Sung KC (2010). Metabolic syndrome, insulin resistance and systemic inflammation as risk factors for reduced lung function in Korean nonsmoking males. J Korean Med Sci.

[10] Kim SK, Hur KY, Choi YH, Kim SW, Chung JH, Kim HK (2010). The Relationship between Lung Function and Metabolic Syndrome in Obese and Non-Obese Korean Adult Males. Korean Diabetes J.

[11] Oda E, Kawai R (2009). A cross-sectional relationship between vital capacity and diabetes in Japanese men. Diabetes Res Clin Pract.

[12] Oda E, Kawai R (2009). A cross-sectional relationship between vital capacity and metabolic syndrome and between vital capacity and diabetes in a sample Japanese population. Environ Health Prev Med.

[13] Garin-Shkolnik T, Rudich A, Hotamisligil GS, Rubinstein M (2014). FABP4 attenuates PPARgamma and adipogenesis and is inversely correlated with PPARgamma in adipose tissues. Diabetes.

[14] Elmasri H, Karaaslan C, Teper Y, Ghelfi E, Weng M, Ince TA (2009). Fatty acid binding protein 4 is a target of VEGF and a regulator of cell proliferation in endothelial cells. FASEB J.

[15] Choi JK, Paek D, Lee JO (2005). Normal predictive values of spirometry in Korean population. Tuberc Respir Dis (Seoul).

[16] Curb JD, Ford C, Hawkins CM, Smith EO, Zimbaldi N, Carter B (1983). A coordinating center in a clinical trial: the Hypertension Detection and Followup Program. Control Clin Trials.

[17] Matthews DR, Hosker JP, Rudenski AS, Naylor BA, Treacher DF, Turner RC (1985). Homeostasis model assessment: insulin resistance and beta-cell function from fasting plasma glucose and insulin concentrations in man. Diabetologia.

[18] Jubber A (2004). Respiratory complications of obesity. Int J Clin Pract.

[19] Ray CS, Sue DY, Bray G, Hansen JE, Wasserman K (1983). Effects of obesity on respiratory function. Am Rev Respir Dis.

[20] Maiolo C, Mohamed EI, Carbonelli MG (2003). Body composition and respiratory function. Acta Diabetol.

[21] Cotes JE, Chinn DJ, Reed JW (2001). Body mass, fat percentage, and fat free mass as reference variables for lung function: effects on terms for age and sex. Thorax.

[22] Lazarus R, Sparrow D, Weiss ST (1997). Effects of obesity and fat distribution on ventilatory function: the normative aging study. Chest.

[23] Santana H, Zoico E, Turcato E, Tosoni P, Bissoli L, Olivieri M (2001). Relation between body composition, fat distribution, and lung function in elderly men. Am J Clin Nutr.

[24] Wannamethee SG, Shaper AG, Whincup PH (2005). Body fat distribution, body composition, and respiratory function in elderly men. Am J Clin Nutr.

[25] Mancuso P (1985). Obesity and lung inflammation. J Appl Physiol.

[26] Wang C (2014). Obesity, inflammation, and lung injury (OILI): the good. Mediat Inflamm.

[27] Gurzu B, Zugun FE, Costuleanu M, Mihaescu T, Carasevici E, Petrescu G (2008). Adipokines involvement in lung function. Rev Med Chir Soc Med Nat Iasi.

[28] Furuhashi M, Fucho R, Gorgun CZ, Tuncman G, Cao H, Hotamisligil GS (2008). Adipocyte/macrophage fatty acid-binding proteins contribute to metabolic deterioration through actions in both macrophages and adipocytes in mice. J Clin Invest.

[29] Furuhashi M, Tuncman G, Gorgun CZ, Makowski L, Atsumi G, Hotamisligil GS. Treatment of diabetes and atherosclerosis by inhibiting fatty-acid-binding protein aP2. Nature. 2007;447(7147):959–65. 10.1038/nature05844 PMC4076119 17554340

[30] Hotamisligil GS, Johnson RS, Distel RJ, Ellis R, Papaioannou VE, Spiegelman BM (1996). Uncoupling of obesity from insulin resistance through a targeted mutation in aP2, the adipocyte fatty acid binding protein. Science.

[31] Maeda K, Cao H, Kono K, Gorgun CZ, Furuhashi M, Uysal KT (2005). Adipocyte/macrophage fatty acid binding proteins control integrated metabolic responses in obesity and diabetes. Cell Metab.

[32] Makowski L, Boord JB, Maeda K, Babaev VR, Uysal KT, Morgan MA (2001). Lack of macrophage fatty-acid-binding protein aP2 protects mice deficient in apolipoprotein E against atherosclerosis. Nat Med.

[33] Kaess BM, Enserro DM, McManus DD, Xanthakis V, Chen MH, Sullivan LM (2012). Cardiometabolic correlates and heritability of fetuin-A, retinol-binding protein 4, and fatty-acid binding protein 4 in the Framingham Heart Study. J Clin Endocrinol Metab.

[34] Stejskal D, Karpisek M (2006). Adipocyte fatty acid binding protein in a Caucasian population: a new marker of metabolic syndrome?. Eur J Clin Invest.

[35] Jeon WS, Park SE, Rhee EJ, Park CY, Oh KW, Park SW (2013). Association of serum adipocyte-specific Fatty Acid binding protein with Fatty liver index as a predictive indicator of nonalcoholic Fatty liver disease. Endocrinol Metab (Seoul).

[36] Lee TH, Jeon WS, Han KJ, Lee SY, Kim NH, Chae HB, et al. Comparison of Serum Adipocytokine Levels according to Metabolic Health and Obesity Status. Endocrinol Metab (Seoul). 2015;30(2):185-94. 10.3803/EnM.2015.30.2.185 PMC4508263 25325281

[37] Lin WY, Yao CA, Wang HC, Huang KC (2006). Impaired lung function is associated with obesity and metabolic syndrome in adults. Obesity (Silver Spring).

[38] Nakajima K, Kubouchi Y, Muneyuki T, Ebata M, Eguchi S, Munakata H (2008). A possible association between suspected restrictive pattern as assessed by ordinary pulmonary function test and the metabolic syndrome. Chest.

