# Supplementary material for: The relationship between serum fatty-acid binding protein 4 level and lung function in Korean subjects with normal ventilatory function
Source: BMC Pulm Med. 2016 Feb 18;16:34. doi: 10.1186/s12890-016-0190-8 (PMC4757985; doi:10.1186/s12890-016-0190-8)
Supplement: Additional file 1: Table S1. — Comparisons of the metabolic components in the lowest quartile and other quartiles of FVC (% pred) among subjects with normal ventilatory function. Table S2. Comparisons of the metabolic components in the lowest quartile and other quartiles of FEV1 (% pred) among subjects with normal ventilatory function. (DOCX 18 kb) [file 12890_2016_190_MOESM1_ESM.docx]

**Additional file 1**

**Table S1.** Comparisons of the metabolic components in the lowest quartile and other quartiles of FVC (% pred) among subjects with normal ventilatory function

|  | 1^st^ Quartile  (≤ 0.993) | 2^nd^, 3^rd^, 4^th^ Quartile  (> 0.993) | *P value* |
| --- | --- | --- | --- |
| Age | 42.06±6.37 | 40.78±6.25 | 0.0673 |
| Sex: male | 58(53.7) | 254(78.64) | < 0.0001 |
| Smoking | 12(11.21) | 66(20.56) | 0.0395 |
| BMI | 23.62±3.32 | 23.99±2.76 | 0.3010 |
| SBP | 110.83±13.33 | 114.37±11.44 | 0.0147 |
| DBP | 71.67±10.28 | 73.59±9.06 | 0.0655 |
| Fasting glucose | 94.06±11.91 | 95.15±12.56 | 0.4309 |
| Total cholesterol | 206.54±40.63 | 202.27±34.38 | 0.3283 |
| Triglyceride | 140.83±166.72 | 138.63±95.94 | 0.8967 |
| HDL-C | 56.41±13.4 | 53.03±10.4 | 0.0181 |
| LDL-C | 117.16±30.84 | 115.53±27.21 | 0.6163 |
| Fasting Insulin | 7.21±4.29 | 6.67±3.28 | 0.2356 |
| Percent body fat | 24.9±5.78 | 22.71±5.24 | 0.0003 |
| WHR | 0.86±0.05 | 0.87±0.04 | 0.4825 |
| HOMA-IR | 1.72±1.26 | 1.57±0.84 | 0.2514 |
| Ln(FABP4) | 2.34±0.46 | 2.18±0.45 | **0.0015** |

FVC, forced vital capacity; BMI, body mass index; SBP, systolic blood pressure; DBP, diastolic blood pressure; HDL-C, high-density lipoprotein cholesterol; LDL-C, low-density lipoprotein cholesterol; WHR, waist-hip ratio; HOMA-IR, homeostasis model assessment-insulin resistance; Ln(FABP4), logarithmized form of fatty-acid binding protein 4

**Table S2.** Comparisons of the metabolic components in the lowest quartile and other quartiles of FEV1 (% pred) among subjects with normal ventilatory function

|  | 1st Quartile  (≤ 0.979) | 2^nd^, 3^rd^, 4^th^ Quartile  (> 0.979) | *P value* |
| --- | --- | --- | --- |
| Age | 41.44±6.18 | 40.99±6.35 | 0.5292 |
| Sex: male | 50(46.3) | 262(81.11) | < 0.0001 |
| Smoking | 13(12.14) | 65(20.18) | 0.0849 |
| BMI | 23.66±3.42 | 23.98±2.73 | 0.3701 |
| SBP | 109.54±12.26 | 114.8±11.67 | 0.0001 |
| DBP | 70.83±10.06 | 73.87±9.07 | 0.0036 |
| Fasting glucose | 93.69±10.64 | 95.28±12.92 | 0.2042 |
| Total Cholesterol | 207.75±37.03 | 201.86±35.65 | 0.1420 |
| Triglyceride | 123.58±159.86 | 144.4±99.2 | 0.2049 |
| HDL-C | 57.07±11.64 | 52.81±11.01 | 0.0007 |
| LDL-C | 119±27.51 | 114.92±28.23 | 0.2067 |
| Fasting Insulin | 6.87±4.11 | 6.78±3.36 | 0.8414 |
| Percent body fat | 25.73±5.4 | 22.43±5.23 | < 0.0001 |
| WHR | 0.86±0.05 | 0.87±0.04 | 0.5617 |
| HOMA-IR | 1.62±1.19 | 1.6±0.87 | 0.8795 |
| Ln(FABP4) | 2.29±0.5 | 2.19±0.44 | **0.0710** |

FEV1, forced expiratory volume in 1 second; BMI, body mass index; SBP, systolic blood pressure; DBP, diastolic blood pressure; HDL-C, high-density lipoprotein cholesterol; LDL-C, low-density lipoprotein cholesterol; WHR, waist-hip ratio; HOMA-IR, homeostasis model assessment-insulin resistance; Ln(FABP4), logarithmized form of fatty-acid binding protein 4
